# Pseudohalogen Chemistry in Ionic Liquids with Non‐innocent Cations and Anions

**DOI:** 10.1002/open.202000252

**Published:** 2020-11-10

**Authors:** Sören Arlt, Kevin Bläsing, Jörg Harloff, Karoline Charlotte Laatz, Dirk Michalik, Simon Nier, Axel Schulz, Philip Stoer, Alrik Stoffers, Alexander Villinger

**Affiliations:** ^1^ Anorganische Chemie Institut fur Chemie Universität Rostock A.-Einstein-Str. 3a 18059 Rostock Germany; ^2^ Materialdesign Leibniz-Institut für Katalyse an der Universität Rostock A.- Einstein-Str. 29a 18059 Rostock Germany; ^3^ Anorganische Chemie Institut für Chemie Philipps-Universität Marburg Hans-Meerwein-Straße 4 35032 Marburg Germany

**Keywords:** pseudohalides, hydrogen bonding, ionic liquids, silicates, borates

## Abstract

Within the second funding period of the SPP 1708 “Material Synthesis near Room Temperature”,which started in 2017, we were able to synthesize novel anionic species utilizing Ionic Liquids (ILs) both, as reaction media and reactant. ILs, bearing the decomposable and non‐innocent methyl carbonate anion [CO_3_Me]^−^, served as starting material and enabled facile access to pseudohalide salts by reaction with Me_3_Si−X (X=CN, N_3_, OCN, SCN). Starting with the synthesized Room temperature Ionic Liquid (RT‐IL) [*n*Bu_3_MeN][B(OMe)_3_(CN)], we were able to crystallize the double salt [*n*Bu_3_MeN]_2_[B(OMe)_3_(CN)](CN). Furthermore, we studied the reaction of [WCC]SCN and [WCC]CN (WCC=weakly coordinating cation) with their corresponding protic acids HX (X=SCN, CN), which resulted in formation of [H(NCS)_2_]^−^ and the temperature labile solvate anions [CN(HCN)_*n*_]^−^ (*n*=2, 3). In addition, the highly labile anionic HCN solvates were obtained from [PPN]X ([PPN]=μ‐nitridobis(triphenylphosphonium), X=N_3_, OCN, SCN and OCP) and HCN. Crystals of [PPN][X(HCN)_3_] (X=N_3_, OCN) and [PPN][SCN(HCN)_2_] were obtained when the crystallization was carried out at low temperatures. Interestingly, reaction of [PPN]OCP with HCN was noticed, which led to the formation of [P(CN)_2_]^−^, crystallizing as HCN disolvate [PPN][P(CN⋅HCN)_2_]. Furthermore, we were able to isolate the novel cyanido(halido) silicate dianions of the type [SiCl_0.78_(CN)_5.22_]^2−^ and [SiF(CN)_5_]^2−^ and the hexa‐substituted [Si(CN)_6_]^2−^ by temperature controlled halide/cyanide exchange reactions. By facile neutralization reactions with the non‐innocent cation of [Et_3_HN]_2_[Si(CN)_6_] with MOH (M=Li, K), Li_2_[Si(CN)_6_] ⋅ 2 H_2_O and K_2_[Si(CN)_6_] were obtained, which form three dimensional coordination polymers. From salt metathesis processes of M_2_[Si(CN)_6_] with different imidazolium bromides, we were able to isolate new imidazolium salts and the ionic liquid [BMIm]_2_[Si(CN)_6_]. When reacting [Mes(*n*Bu)Im]_2_[Si(CN)_6_] with an excess of the strong Lewis acid B(C_6_F_5_)_3_, the voluminous adduct anion {Si[CN⋅B(C_6_F_5_)_3_]_6_}^2−^ was obtained.

## Graphical abstract

**The reaction of ionic liquids** (ILs) with methylcarbonate anions like [R_3_MeE][CO_3_Me] (E=N: R=Et, *n*Pr, *n*Bu; E=P, R=Ph) with Me_3_Si−X (X=CN, N_3_, OCN, SCN) or H_2_SiF_6_ led to quantitative formation of pseudohalide salts [R_3_MeE]X or fluorosilicates, e. g. [R_3_MeN]_2_[SiF_6_]. From these precursors, novel anionic species like [B(OMe)_3_(CN)]^−^ or cyanido(halido)silicates of the type [SiCl_0.78_(CN)_5.22_]^2−^, [SiF(CN)_5_]^2−^ and [Si(CN)_6_]^2−^ were obtained. Functionalization of the hexacyanidosilicate with the Lewis acid B(C_6_F_5_)_3_ led to the voluminous WCA {Si[CN⋅B(C_6_F_5_)_3_]_6_}^2−^. Beside [H(NCS)_2_]^−^, highly labile solvate anions of the type [X(HCN)_2_]^−^ (X=CN, SCN), [X(HCN)_3_]^−^ (X=CN, N_3_, OCN) and [P(CN⋅HCN)_2_]^−^ were obtained from IL‐like mixtures, when starting from [WCC]X ([WCC]=[Ph_4_P], [PPN]) and neat HCN.

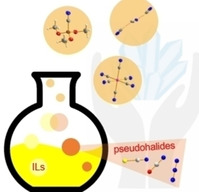

## 1 Introduction

With this Minireview, we would like to summarize our results obtained during the 2nd period of the SPP 1708 “Material Synthesis near Room Temperature”. SPP 1708 was mainly set up to identify and demonstrate the advantages (but also the disadvantages) of ILs when used as reaction media for synthesis in contrast to common inorganic and organic solvents. In synthesis, ILs can play an active role as template, *e. g*. for the precipitation of nanoparticles,[^1^, ^2^, ^3^, ^4^] or it is more or less inert providing a highly polar reaction media composed of ions.^[5]^ Even after six years of SPP 1708, utilization of ILs as reaction media for the synthesis of inorganic compounds is still a fairly new area steadily growing and with the focus on metal/metal oxide/metal nitride/metal halide (nano)particles of different types,[^6^, ^7^, ^8^, ^9^, ^10^] semiconductors,^[11]^ and inorganic solids (*e. g*. zeolites).[^12^, ^13^, ^14^, ^15^, ^16^, ^17^, ^18^, ^19^, ^20^, ^21^, ^22^, ^23^, ^24^] Besides application in classic solid state chemistry, ILs can be used as reaction media to prepare (oligomeric) metal complexes, metal organic frameworks, coordination polymers[^25^, ^26^, ^27^, ^28^, ^29^, ^30^, ^31^, ^32^, ^33^, ^34^, ^35^] or cluster compounds incorporating main group elements.[^36^, ^37^, ^38^, ^39^, ^40^, ^41^] Within SPP 1708, the synthesis of intermetallic cluster and nanoparticles,[^3^, ^42^, ^43^, ^44^, ^45^, ^46^, ^47^, ^48^, ^49^, ^50^, ^51^, ^52^, ^53^, ^54^, ^55^, ^56^] the controlled synthesis of polyanions and cations,[^57^, ^58^, ^59^, ^60^, ^61^] solvent‐free chalcogenidometal‐containing materials,[^62^, ^63^, ^64^, ^65^, ^66^, ^67^, ^68^, ^69^, ^70^, ^71^, ^72^, ^73^, ^74^, ^75^] deposition of nanocrystalline materials,[^76^, ^77^, ^78^, ^79^, ^80^] ionic liquids as precursors for inorganic materials,[^81^, ^82^, ^83^] ionic‐liquid‐modified hybrid materials,[^84^, ^85^, ^86^, ^87^] as well as the low‐temperature synthesis of thermoelectric materials[^88^, ^89^, ^90^, ^91^, ^92^] were investigated. Moreover, theoretical[^93^, ^94^, ^95^, ^96^, ^97^, ^98^, ^99^, ^100^, ^101^, ^102^, ^103^, ^104^, ^105^, ^106^] and solubility[^107^, ^108^, ^109^, ^110^, ^111^, ^112^, ^113^, ^114^] aspects during the synthesis process were studied. Among the publications with respect to main group molecule chemistry, some highlights are the isolation and characterization of [P_3_Se_4_]^+^ by Ruck et al.,^[115]^ the cluster anions of the type [M_4_Sn_4_Se_17_]^10−^ (M=Mn, Zn, Cd) by Dehnen et al.^[72]^ and the isolation of elusive hydrogen bonded poly(hydrogen halide) halogenates [X(HY)]^−^ (X=Br, I, ClO_4_^−^; Y=Cl, Br, CN) by Hasenstab‐Riedel and coworker.^[116]^

Following our interests in pseudohalide chemistry, we focused on the synthesis of small molecules with the primary goal to make very simple isolable compounds. During the first funding period we were able to synthesize and characterize salts bearing new heteroleptic cyanido(fluorido)phosphate anions of the general formula [PF_6−*n*_(CN)_*n*_]^−^ (*n*=1−4),[^117^, ^118^] as well as the homoleptic tetracyanidoborate anion [B(CN)_4_]^−^,^[119]^ by very mild Lewis‐acid catalyzed synthesis protocols, when starting from ILs. We succeeded in the synthesis and structural characterization of molecular pnictogen tricyanides E(CN)_3_ (E=Sb, Bi), which were stabilized in the IL [BMIm][OTf] ([BMIm]=1‐*n*Butyl‐3‐methylimidazolium, [OTf]=F_3_CSO_2_O^−^), thus preventing these species from oligomerization.^[120]^ Utilizing ILs, we were able to synthesize salts with homoleptic [E(CN)_5_]^2−^ anions (E=Sb, Bi), but never formed any higher substituted species like [Bi(CN)_6_]^3−^ or [Bi_2_(CN)_11_]^5−^, which we obtained when using common organic solvents such as acetonitrile.^[121]^ Our attempts in synthesizing cyanidoarsenates of the type [As(CN)_3+*n*_]^*n*−^ (*n*=1−3) always led to formation of an isomer of [As(CN)_4_]^−^, the arsazolide heterocycle [AsC_4_N_4_]^−^, regardless of whether we used ILs or organic solvents like acetonitrile.^[122]^

Here, we mainly want to summarize on novel pseudohalide‐containing anions that were obtained using ILs as reaction media.

## 2 Synthesis of Pure Pseudohalide Containing ILs from ILs with Decomposable Anions[^123^, ^124^, ^125^]

**Scheme 1:**
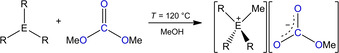
Synthesis of methylcarbonates with trialkylmethylammonium (E=N, R=Et, *n*Pr, *n*Bu) or methyltriphenylphosphonium (E=P, R=Ph) counter ions in methanol with dimethyl carbonate and a tertiary alkylated amine or triphenylphosphine, respectively.

We started this project with the synthesis of various trialkylmethylammonium‐ ([R_3_MeN]: R=Et, *n*Pr, *n*Bu)[^123^, ^124^] and methyltriphenylphosphonium methylcarbonates.^[125]^ They were formed in autoclave reactions at autogenous pressure (Scheme 1), due to a modified reaction, which was first described by Werntz^[126]^ and taken up again in later years by various working groups.[^127^, ^128^, ^129^]

**Scheme 2:**
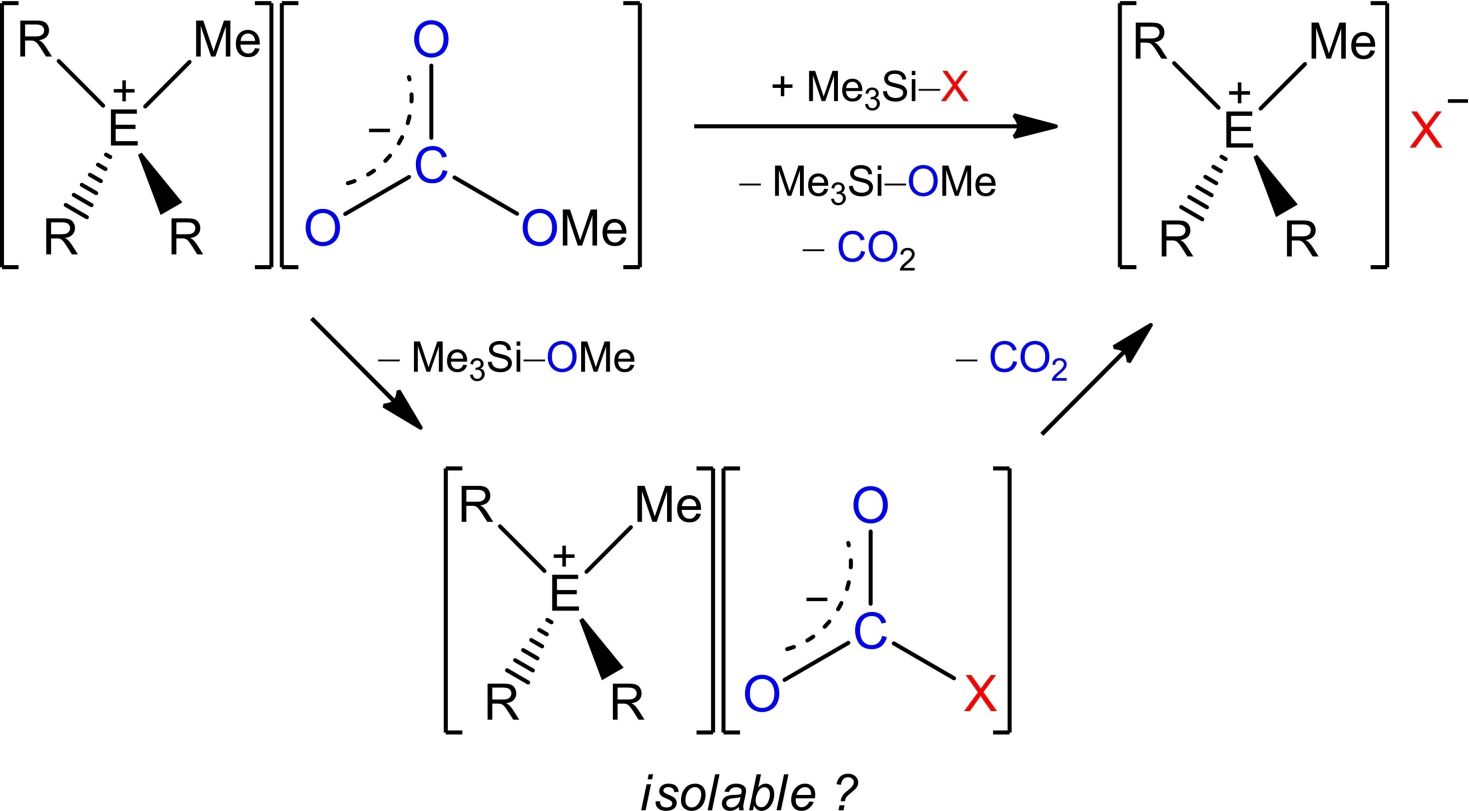
Formation of trialkylmethylammonium (E=N, R=Et, *n*Bu with X=CN, N_3_, OCN, SCN; E=N, R=*n*Pr with X=CN) or triphenylmethyl‐phosphonium (E=P, R=Ph with X=CN) pseudohalides via nucleophilic desilylation of a pseudohalogen trimethylsilane by methylcarbonate‐containing salts under formation of a pseudohaloformate anion as possible intermediate.

In a subsequent reaction we synthesized the pseudohalides of the type [R_3_MeN]X (R=Et, *n*Bu: X=CN, N_3_, OCN, SCN;^[123]^ R=nPr: X=CN)^[124]^ and [Ph_3_MeP]CN,^[125]^ by nucleophilic desilylation of trimethylpseudohalosilanes Me_3_Si–X with the reactive and non‐innocent [CO_3_Me]^−^ anion (Scheme 2), a reaction developed by Sundermeyer et al.^[129]^ Besides the pseudohalide salt, Me_3_Si–OMe and CO_2_ were formed, whereby the latter can be removed from the reaction solution, which shifted the reaction equilibrium in favor of the products. The formation of the stable Si−O bond (444 kJ/mol)^[130]^ is also a driving force of this reaction. These synthesized ILs (and salts with a low‐temperature melting point), containing methyl carbonates and pseudohalides, provided the basis for the majority of all planned reactions, which are described in the following sections.

In addition to the synthesis of the pseudohalide salts, we aimed to isolate possible intermediates such as [CO_2_X]^−^ (X=pseudohalogen), which are presumably formed during the decomposition reaction (Scheme 2). The fluoro‐ and cyanoformates [CO_2_F]^−[131]^ or [CO_2_CN]^−[132]^ are already literature known, of which the latter was synthesized as the [Ph_4_P]^+^ salt by exposure of CO_2_ to a concentrated solution of [Ph_4_P]CN in acetonitrile.

**Scheme 3:**
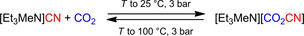
Temperature dependant equilibrium of cyanoformate and the starting materials [Et_3_MeN]CN and CO_2_ at a CO_2_ pressure of 3 bar.

First, we tried to isolate pseudohaloformate anions from concentrated and cooled reaction solutions of [R_3_MeN][CO_3_Me] and Me_3_Si−X in acetonitrile.^[133]^ But even at −40 °C the release of gaseous CO_2_ was observed and no new signals for any [CO_2_X]^−^ species could be observed by means of ^13^C{^1^H} NMR spectroscopy. Subsequently, a solvent free process was used, from which the [CO_2_CN]^−^ anion was synthesized with [Et_3_MeN]^+^ as counter cation, when heating pure [Et_3_MeN]CN to 150 °C in a CO_2_ atmosphere at a pressure of 3 bar. Initially, the salt remained solid at high temperature but then liquefied when it was cooled down to room temperature. Raman spectroscopy revealed that at room temperature the RT‐IL [Et_3_MeN][CO_2_CN] with *ν*_CN_=2196 cm^−1^ is present, while at elevated temperature (100 °C) C−C bond cleavage (activation barrier ∼40 kJ/mol)^[132]^ and formation of the starting materials [Et_3_MeN]CN with *ν*_CN_=2049 cm^−1^ and CO_2_ is observed (Scheme 3). In a closed system this process is reversible, whereby [Et_3_MeN][CO_2_CN] is formed again upon cooling. However, when the system is opened, CO_2_ is irreversibly released and [Et_3_MeN]CN is formed.

Nevertheless, a reaction of other ammonium pseudohalides of the type [*n*Bu_3_MeN]X (X=N_3_, OCN und SCN) with CO_2_ could not be observed under similar reaction conditions.

## 3 Reactions of Pseudohalides Containing ILs with P_4_ and P_4_S_10_ ^[133]^

**Scheme 4:**
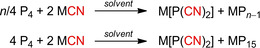
Synthesis of dicyanphosphides and polyphosphosphides by nucleophilic degradation of white phosphorus with cyanides (M=[18]crown‐6‐Na/K, [Et_4_N], [*n*Bu_4_N], [PPN]).^[134]^

As Schmidtpeter et al. could show, white phosphorus is degraded by cyanide salts to dicyanophosphide and different polyphosphides in solution (Scheme 4).^[134]^

We wondered whether new phosphorus pseudohalide compounds could be synthesized, using the pure ILs without further solvents.^[133]^ To prevent the white phosphorus from sublimating, a mixture of P_4_ and the ILs [*n*Bu_3_MeN]X (X=CN or N_3_) was filled into an ampoule. This was sealed and then heated to 65 °C (X=N_3_) and 105 °C (X=CN), respectively, causing the solids to liquefy. When heating the mixture for 24 h, a shiny violet‐black solid was formed in both reaction vessels and a red (X=N_3_) or colorless (X=CN) liquid was formed as well. Subsequently, the reaction products were extracted with acetonitrile and benzene, leaving an insoluble black solid. By means of ^1^H and ^13^C{^1^H} NMR spectroscopic analysis of the liquid phase, the decomposition of the cation to *n*Bu_3_N could be determined in both reactions. The reaction of P_4_ with cyanide also resulted in the formation of traces of [P(CN)_2_]^−^ as was observed by Schmidtpeter et al.^[134]^ The solid residues could not be examined further by Raman spectroscopy due to fluorescence, but elemental analyses of the substances indicated a high phosphorus content of the compound with 61 % for X=N_3_ and 33.7 % for X=CN. The formation of further unknown pseudohalide phosphorus compounds could not be observed by means of ^31^P NMR spectroscopy. Due to the instability of the cation, no further experiments with [*n*Bu_3_MeN]X (X=OCN and SCN) were performed.

**Scheme 5:**
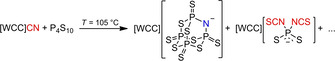
Reaction of phosphorus pentasulfide with [WCC]CN ([WCC]=[*n*Bu_3_MeN]) under formation of the [P_4_S_9_N]^−^ and [(SCN)_2_PS_2_]^−^ anions.

Likewise, the aim was to investigate whether the pseudohalide salts undergo reaction with phosphorus pentasulfide, hopefully leading to the formation of new compounds via a solvent‐free synthesis process. Roesky et al. already observed the formation of [(NCPS_2_)_2_S]^−^, [(N_3_)_2_PS_2_]^−^ and [(N_3_PS_2_)_2_S]^−^ or [(SCN)_2_PS_2_]^−^, which were isolated as [*n*Pr_4_N] salts by reaction of MX (M=Na or K; X=CN, N_3_ or SCN) with P_4_S_10_ in acetonitrile.^[134]^ We heated a mixture of two equivalents of [*n*Bu_3_MeN]CN with one equivalent of P_4_S_10_ at 105 °C for 24 h, thus resulting in liquefaction of both reactants and formation of a homogeneous phase. In solvents such as Et_2_O, benzene, thf or *n*‐hexane, no formed products could be extracted, whereas in acetonitrile the complete residue was dissolved. From the complex reaction mixture, only two formed products could be assigned by ^31^P NMR and (ESI‐TOF)‐MS. One was the adamantane‐like [P_4_S_9_N]^−^ anion[^135^, ^136^] (*δ*[^31^P]=67 and 33 ppm; m/z=426) and the second was the [(SCN)_2_PS_2_]^−^ anion^[134]^ (*δ*[^31^P]=53 ppm, m/z=211), which are already known from literature and are shown in Scheme 5. Interestingly, Roesky et al. prepared the [P_4_S_9_N]^−^ anion by the reaction of [(N_3_)_2_PS_2_]^−^ with P_4_S_10_, while the [(SCN)_2_PS_2_]^−^ was formed from P_4_S_10_ and KSCN.

As the products could neither be extracted from the mixture nor crystallized in different mixtures of solvents, *e. g*. acetonitrile/benzene or acetonitrile/diethyl ether, no further investigations with other ILs [nBu_3_MeN]X (X=N_3_, OCN, SCN) were carried out.

## 4 Reactions of Pseudohalides containing ILs with Acids of Pseudohalides (HA)[^125^, ^137^, ^138^]

**Scheme 6:**
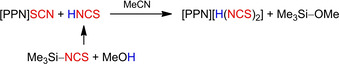
Synthesis of hydrogen dithiocyanate from the reaction of [PPN]SCN with *in situ* generated HNCS.

When attempting the synthesis of [PPN]SCN from [PPN]Cl and KSCN in H_2_O, we surprisingly obtained crystals of [PPN][H(NCS)_2_].^[137]^ A direct method for the synthesis of this compound was achieved when [PPN]SCN was treated with *in situ* formed HNCS, generated from MeOH and Me_3_Si−NCS (Scheme 6).

**Figure 1:**
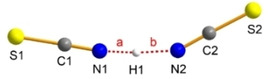
Ball‐and‐stick representation of the [H(NCS)_2_]^−^ ion in the crystal of [PPN][H(NCS)_2_]. Dashed red lines (a and b) represent hydrogen bridges.

According to X‐ray analysis, a slightly bent anion is formed, with one N⋅⋅⋅H⋅⋅⋅N hydrogen bridge (Figure 1, contacts a and b). Dove and Nuzzo et al. already observed the formation of molecular [H(NCS)_2_]^−^ ion when a solution of [WCC]SCN ([WCC]=[Ph_4_As], [Ph_4_P]) was treated with the acid HNCS.[^139^, ^140^]

**Scheme 7:**
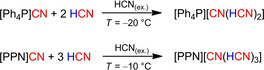
Synthesis of dihydrogen tricyanide and trihydrogen tetracyanide containing salts by reaction of [Ph_4_P]CN or [PPN]CN with liquid HCN.

Already in 1978, Salthouse and Waddington observed that, when HCN was added to [*n*Pr_4_N]CN, the solution became increasingly viscous due to polymerization of the HCN and also noticed that the color of the solution quickly intensified. Crystals, which formed in the black oil, were examined by IR spectroscopy. A single band for *ν*_CN_=2060 cm^−1^ indicated the formation of hydrogen dicyanide [H(CN)_2_]^−^, but a solid state structure was not determined.^[141]^ Thus motivated, we tried to synthesize salts containing the [CN(HCN)_*n*_]^−^ (*n*=1, 2, 3 …) ions by reaction of pure HCN with [WCC]CN ([WCC]=[Et_3_MeN], [*n*Pr_3_MeN], [*n*Bu_3_MeN], [Ph_3_MeP]).^[125]^ In an initial study the salts were suspended in Me_3_Si–CN and then MeOH was added to generate HCN *in situ*, but no crystallization could be achieved when cooling the solutions. Therefore, the [WCC]CN were dissolved directly in an excess of 15 equivalents of HCN, which was cooled to 0 °C. The concentrated, highly ionic solution with the characteristics of an IL turned from yellow to brown within an hour and became increasingly viscous, which can be explained by the formation of polymeric HCN species.^[142]^ Since in all cases no crystallization of a product could be achieved, different salts of the type [WCC]CN ([WCC]=[PPN]), [Ph_4_P],) were used, which contain symmetrical cations and thus crystallize faster (Scheme 7).

**Figure 2:**
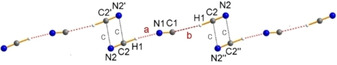
Ball‐and‐stick representation of a section of [CN(HCN)_2_]^−^ anion strands in the crystal of [Ph_4_P][CN(HCN)_2_]. Red dashed lines (a and b) show hydrogen bonding whereas the grey dashed lines (c) show weak Van der Waals interactions.

Colorless crystals of [Ph_4_P][CN(HCN)_2_] could be obtained from a solution of [Ph_4_P]CN in pure liquid HCN, when the mixture was slowly cooled to −20 °C. The compound is sensitive to temperature as well as moisture and decompose over time, observed by Raman spectroscopy. X‐ray structure analysis proved the formation of molecular dihydrogen tricyanide anions [CN(HCN)_2_]^−^ (Figure 2). The anions form distorted chains which are formed by hydrogen bonds (Figure 2, contacts a and b). The chains are connected to each other by weak Van der Waals interaction (Figure 2, contact c) by a head‐tail arrangement and thus form chains along the *a*‐axis in the crystal.

Due to disorder, three other isomers can be considered besides the isomer [NC–H⋅⋅⋅CN⋅⋅⋅H−CN]^−^ (*P*=73 %, major), which show the formation of hydrogen isocyanide ([CN−H⋅⋅⋅CN⋅⋅⋅H−CN]^−^, [NC−H⋅⋅⋅CN⋅⋅⋅H−NC]^−^, [CN−H⋅⋅⋅CN⋅⋅⋅H−NC]^−^). DFT calculations (pbe0/aug‐cc‐pVTZ) support this finding, since the thermodynamically preferred isomer also has the lowest energy of all four calculated linear anions. Furthermore, the energies for a rotation of the central cyanide ion in a flexible [CN(HCN)_2_]^−^ system were calculated. An activation energy of 7.0 kcal/mol is low enough to allow the central anion to rotate freely in solution at room temperature, thus explaining a disorder.

**Figure 3:**
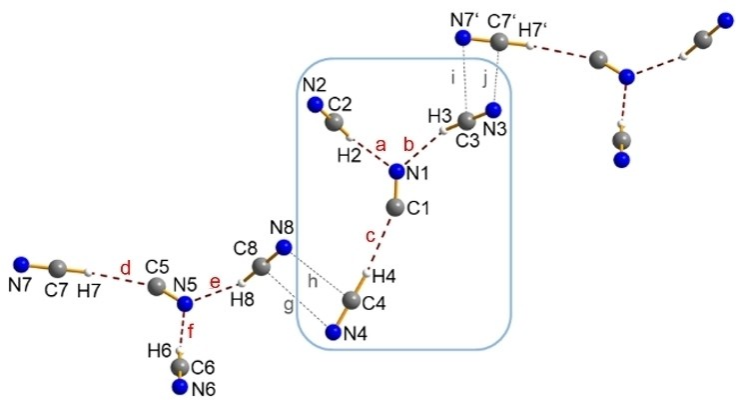
Ball‐and‐stick representation of a section of molecular Y‐shaped [CN(HCN)_3_]^−^ anions in the crystal of [PPN][CN(HCN)_3_]. Red dashed lines (a–f) show hydrogen bonding whereas the grey dashed lines (g–j) show weak Van der Waals interactions.

When [PPN]CN was used instead of [Ph_4_P]CN, crystals with the trihydrogen tetracyanide ion [PPN][CN(HCN)_3_] are formed. Intra‐ionic hydrogen bonds (Figure 3, contacts a–f) and inter‐ionic Van der Waals interactions (Figure 3, contacts g–j) also lead to chains along the *b*‐axis in the crystal. Y‐shaped molecular anions are built, due to the larger cavities formed by the bulky [PPN] cation, which allows the aggregation of a further HCN molecule. This Y‐shaped isomer, which contains only hydrogen cyanide molecules, also represents the energetically preferred structure of all calculated isomers of the trisolvates.

**Figure 4:**
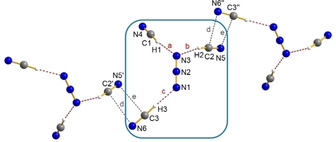
Ball‐and‐stick representation of a section of molecular Y‐shaped [N_3_(HCN)_3_]^−^ anions in the crystal of [PPN][N_3_(HCN)_3_]. Red dashed lines (a–c) show hydrogen bonding whereas the grey dashed lines (d and e) show head‐to‐tail contacts.

**Figure 5:**
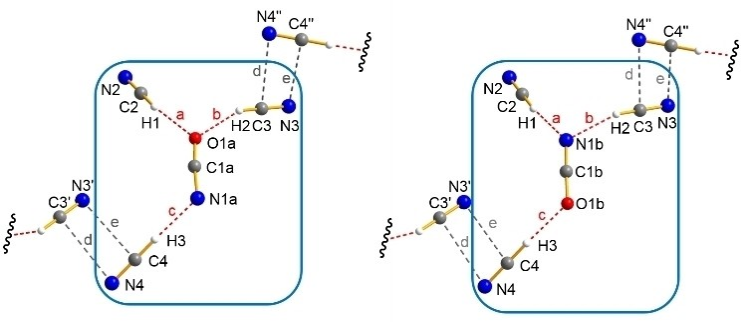
Ball‐and‐stick representation of a section of molecular Y‐shaped [OCN(HCN)_3_]^−^ anions in the crystal of [PPN][OCN(HCN)_3_]. Two isomers are formed due to disorder (left: *a*‐layer 52(1) %, right: *b*‐layer 48(1) %). Red dashed lines (a–c) show hydrogen bonding whereas the grey dashed lines (d and e) show head‐to‐tail contacts.

**Figure 6:**
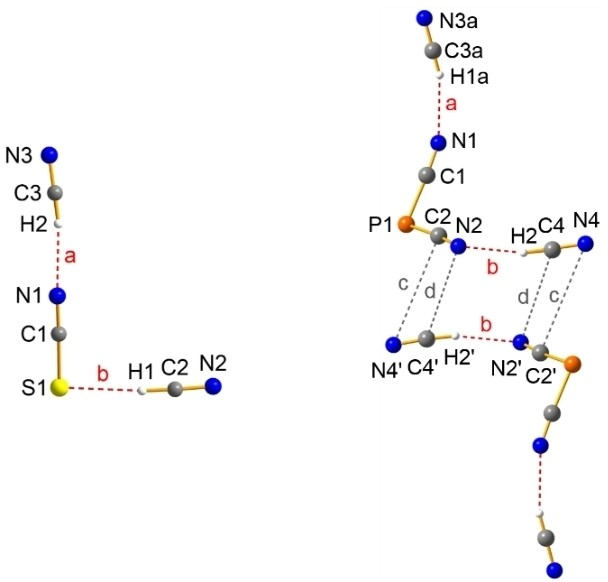
Left: Ball‐and‐stick representation of the molecular L‐shaped [SCN(HCN)_2_]^−^ ion in the crystal of [PPN][SCN(HCN)_2_]. Right: Ball‐and‐stick representation of the molecular [P(CN⋅HCN)_2_]^−^ ion in the crystal of [PPN][P(CN⋅HCN)_2_]. Red dashed lines (a and b) show hydrogen bonding whereas the grey dashed lines (c and d) show head‐to‐tail contacts.

In addition, we were curious, whether it is possible to stabilize and isolate HCN aggregates with other pseudohalides, such as azide, cyanate, thiocyanate and the phosphaethynolate anion in form of their [PPN]^+^ salts.^[138]^ Again, the pseudohalides were dissolved in cooled HCN, but unfortunately no crystals could be obtained from the concentrated and cooled phase. Hence, a rather unusual crystallization method was used. Fomblin YR‐1800 perflouropolyether was placed in a low‐temperature stage for crystal picking, which was cooled to −60 °C. The HCN salt mixtures were cooled until the liquid layer of the IL‐like mixture slowly solidified and subsequently, small portions of the solid were transferred into the ether. Even at −60 °C, the release of gaseous HCN could be observed which resulted in formation of X‐ray suitable crystals. The azide and cyanate salts crystallized as trisolvates [PPN][X(HCN)_3_] (X=N_3_, OCN) (Figure 4 and Figure 5). Both molecular anions exhibit a distorted Y‐shaped geometry. Head‐to‐tail contacts of adjacent anions can be observed but with quite long interionic distances. Hence, the formation of anionic strands, which are located in the cavities formed by the bulky [PPN]^+^ ions, are better explained by packing effects in the crystal. Due to disorder of the central cyanate anion in [OCN(HCN)_3_]^−^, two different isomers have to be considered. On the one hand, an isomer (52(1) %) in which two C−H⋅⋅⋅O and one C−H⋅⋅⋅N H‐bridges are observed (Figure 5, left), and on the other hand an isomer (48(1) %) in which two C−H⋅⋅⋅N and one C−H⋅⋅⋅O H‐bridges are found (Figure 5, right). The thiocyanate salt crystallized as HCN disolvate [PPN][SCN(HCN)_2_] forming a L‐shaped molecular anion with one C−H⋅⋅⋅N and one C−H⋅⋅⋅S hydrogen bridge (Figure 6, left). Computations suggest that the energetically favored isomers exhibit a Y‐shaped structure with two C−H⋅⋅⋅N H‐bridges. However, the observed L‐shaped geometry was found to be only 0.58 kcal/mol higher in energy. Interestingly, if the pseudohalide PCO^−^,^[143]^ which was synthesized in form of [PPN]PCO, is dissolved in HCN, the degradation of the phosphaethynolate anion was observed. This led to the formation of the dicyanophosphide anion [P(CN)_2_]^−^, which crystallized as HCN disolvate [P(CN⋅HCN)_2_]^−^ (Figure 6, right).

## 5 Synthesis of Pseudohalogen Borate and Phosphate Species in Pseudohalide Containing ILs^[123]^

**Scheme 8:**
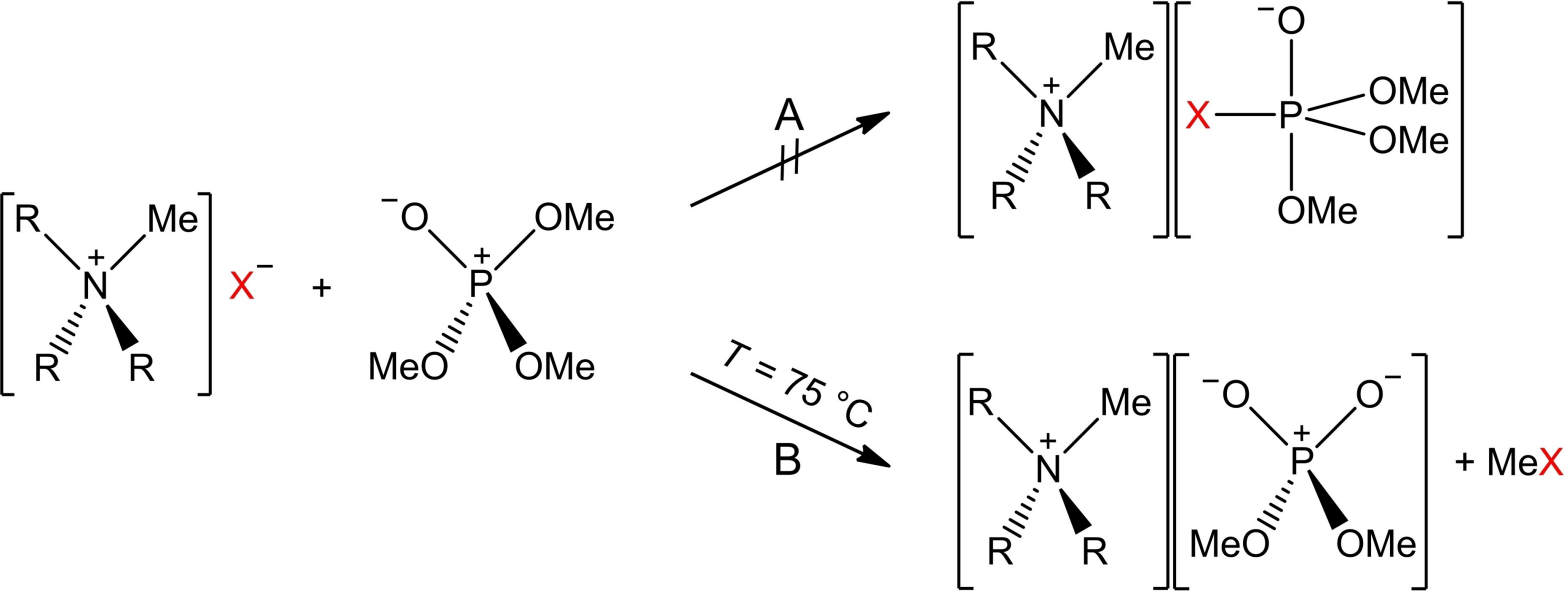
Route A: Assumed nucleophilic addition of ammonium pseudohalides (R=*n*Bu, X=CN, N_3_, OCN, SCN) to trimethoxy phosphate. Route B: Observed reaction of the ammonium pseudohalides with OP(OMe)_3_ and formation of ammonium dimethylphosphates via methylation of the pseudohalides.

The Lewis acidities of trimethyl phosphate OP(OMe)_3_ and trimethyl borate B(OMe)_3_ were investigated towards the pure ILs [*n*Bu_3_MeN]X (X=CN, N_3_, OCN, SCN), hopefully leading to new pseudohalide phosphates or borates. However, a reaction of [*n*Bu_3_MeN]X with OP(OMe)_3_ did not lead to the formation of new pseudohalide phosphates as hoped (Scheme 8, route A), but in all cases to methylation of the pseudohalide and to formation of [*n*Bu_3_MeN][O_2_P(OMe)_2_], which could be shown clearly by means of NMR spectroscopy (Scheme 8, route B).^[123]^

**Scheme 9:**
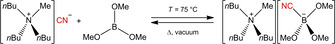
Synthesis of the RT‐IL [*n*Bu_3_MeN][B(OMe)_3_(CN)] and decomposition of the anion into its starting materials at elevated temperature in vacuum.

When heating [*n*Bu_3_MeN]X and B(OMe)_3_ in a flask, a reaction was observed exclusively with X=CN. When heating an equimolar mixture slowly to 75 °C, the RT‐IL [*n*Bu_3_MeN][B(OMe)_3_(CN)], with a melting point of −51 °C, was formed. The cyanidotrimethoxyborate anion is thermally unstable and can be transferred to the starting materials in vacuum, which is accelerated when raising the temperature (Scheme 9). After cooling the RT‐IL to room temperature, the formation of crystals could be observed. X‐ray elucidation of the single crystals revealed the formation of the double salt [*n*Bu_3_MeN]_2_{[B(OMe)_3_(CN)](CN)}, with the novel [B(OMe)_3_CN]^−^ ion. According to a patent by Finze et al., the formation of [B(OMe)_3_(CN)]^−^ has only been observed as intermediate by means of NMR and MS analysis, when synthesizing [B(OMe)_4−*n*_(CN)_*n*_]^−^ (*n*=2, 3), starting from [B(OMe)_4_]^−^ and cyanotrimethylsilane.^[144]^

## 6 Reaction of Pseudohalogen Borate ILs with Persilylated Compounds of Group 15^[133]^

**Scheme 10:**
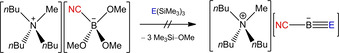
Assumed reaction of [*n*Bu_3_MeN][B(OMe)_3_(CN)] with E(SiMe_3_)_3_ (E=N, P) via Me_3_Si–OMe elimination reactions.

Since it is already known that [B(OMe)_4_]^−^ undergoes reaction with cyanotrimethylsilane to form [B(OMe)_4−*n*_(CN)_*n*_]^−^ (*n*=2, 3) under elimination of Me_3_Si−OMe, the reactivity of the synthesized [B(OMe)_3_(CN)]^−^ anion towards tris(trimethylsilyl)phosphane and tris(trimethylsilyl)amine was investigated.^[133]^ The aim was to synthesize new cyanide‐containing compounds by the same silylether elimination reaction under formation of a boron‐element bond (Scheme 10).

An equimolar mixture of [*n*Bu_3_MeN][B(OMe)_3_(CN)] and E(SiMe_3_)_3_ (E=N, P) was placed in a flask. In both cases, a two‐phase system was formed in which the starting materials remained unchanged at room temperature, showing no reaction. When heating the mixtures to 55 °C, no reaction with N(SiMe_3_)_3_ was observed according NMR analysis. However, with P(SiMe_3_)_3_ partial decomposition of the ammonium cation to *n*Bu_3_N and formation of Me_3_Si−OMe and B(OMe)_3_ was found, accompanied with the formation of [B(OMe)_2_(CN)_2_]^−^, which could be observed by means of (ESI‐TOF)‐MS. According to ^11^B and ^31^P NMR, a desired product synthesis with B−P bond formation did not occur.

## 7 Synthesis of Coordination Polymers Utilizing Cyanide Silicate Containing ILs with Decomposable Cations[^124^, ^145^]

**Scheme 11:**
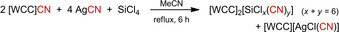
Reaction of [WCC]CN and AgCN with SiCl_4_ ([WCC]=[*n*Pr_3_MeN], [Ph_4_P]).

The synthesis of hexacyanidosilicate dianions [Si(CN)_6_]^2−^ was motivated by the fact that this anion, besides the already synthesized pseudohalide analogues [SiX_6_]^2−^ (X=N_3_,[^146^, ^147^] OCN,[^148^, ^149^] SCN,[^150^, ^151^] SeCN,^[152]^ NCCrCo_5_ ^[153]^), was not yet known. The first attempt to synthesize hexacyanido silicates was performed via Cl/CN substitution reactions.^[145]^ Heating a mixture of SiCl_4_, AgCN and [WCC]CN ([WCC]=[*n*Pr_3_MeN] or [Ph_4_P]) in acetonitrile, led to incomplete chloride/cyanide exchange. We were able to isolate [Ph_4_P]_2_[SiCl_0.78_(CN)_5.22_] as well as [*n*Pr_3_MeN][Ag(CN)Cl] from the corresponding mixtures (Scheme 11, Figure 3). Portius et al. faced the same problem when synthesizing hexacyanidosilicates as they could show in their publication, which appeared at the same time as ours.^[154]^ Hence, we thought, that utilization of IL‐like reaction mixtures could help to solve this problem.

**Scheme 12:**
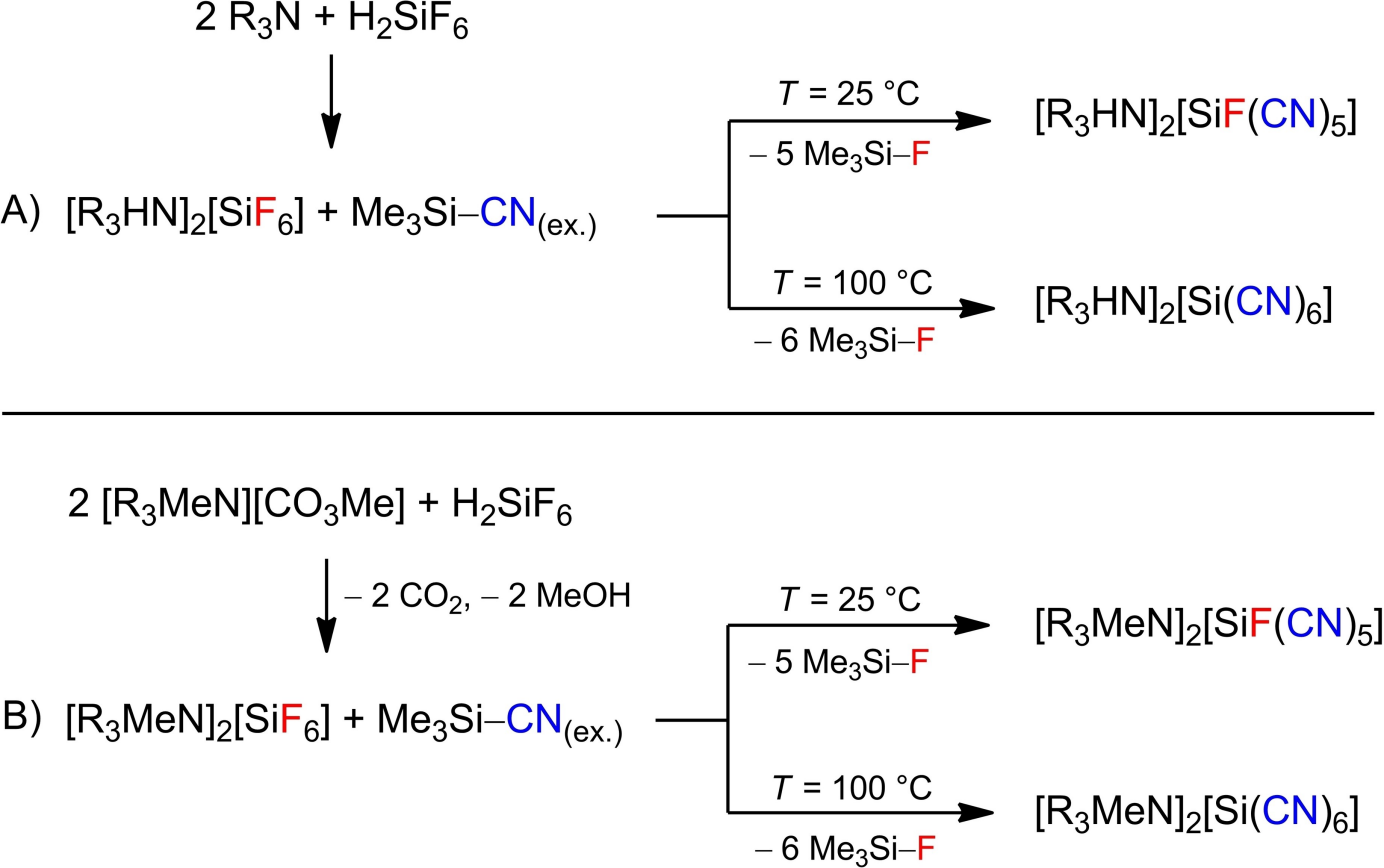
Synthesis of [R_3_HN]_2_[SiF_6_] and [R_3_MeN]_2_[SiF_6_] as well as their conversion into [R_3_HN]_2_[SiF(CN)_5_]/[R_3_HN]_2_[Si(CN)_6_] and [R_3_MeN]_2_[SiF(CN)_5_]/ [R_3_MeN]_2_[Si(CN)_6_] (R=Et, *n*Pr and *n*Bu). For reasons of clarity, the [SiF_5_]^−^ impurities in the starting material were omitted.

**Figure 7:**
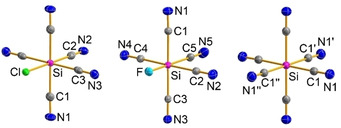
ORTEP representation of the molecular anion structure in the crystal of [Ph_4_P]_2_[SiCl_0.78_(CN)_5.22_] ⋅ 4 MeCN (left), [*n*Pr_3_HN]_2_[SiF(CN)_5_] (middle) and [*n*Pr_3_HN]_2_[Si(CN)_6_] (right). Cations and solvent molecules are omitted for clarity. Thermal ellipsoids are shown at 50 % level of probability.

Therefore, we changed the synthesis strategy. We started with the synthesis of [R_3_HN]_2_[SiF_6_] (R=Et, *n*Pr), by protonation of tertiary amines with H_2_SiF_6_ (Scheme 12, route A), and [R_3_MeN]_2_[SiF_6_] (R=*n*Pr, *n*Bu), which were formed by decomposition reactions of H_2_SiF_6_ with the previously synthesized ILs, the ammonium methylcarbonates (Scheme 12, route B). Subsequently, the fluorido silicates were suspended in 20 equivalents of Me_3_Si−CN and selectively converted to [SiF(CN)_5_]^−^ and [Si(CN)_6_]^−^ by temperature‐controlled F/CN substitution reactions (Scheme 12, Figure 7), which could be isolated on a preparative scale. Catalytic amounts of GaCl_3_ as Lewis acid shortened the reaction time when synthesizing the hexacyanidosilicate dianions.

**Scheme 13:**
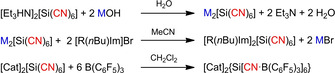
Top: Synthesis of M_2_[Si(CN)_6_] (M=Li, K) via neutralization reactions. Middle: Synthesis of imidazolium hexacyanidosilicates via salt metathesis reactions (R=2‐MePh, 4‐MePh, 2,4,6‐MePh=Mes, 2‐MeOPh, 2,4‐FPh, 4‐BrPh). Bottom: Synthesis of the fully functionalized Lewis acid‐base adduct anion {Si[CN⋅B(C_6_F_5_)_3_]_6_}^2−^ with [Cat]=[Mes(*n*Bu)Im] as counter ion.

When using decomposable cations such as [Et_3_HN]^+^, we were able to synthesize M_2_[Si(CN)_6_] (M=Li, K) by facile neutralization reactions of [Et_3_HN]_2_[Si(CN)_6_] with LiOH or KOH (Scheme 13, top). X‐ray analysis of Li_2_[Si(CN)_6_] ⋅ 2 H_2_O and K_2_[Si(CN)_6_] revealed the formation of coordination polymers, forming a 3D network in both cases. Subsequently, the metal salts were transferred by metathesis reactions with [BMIm]Br^[124]^ (1‐Butyl‐3‐methylimidazolium) or *TAAILs*[^155^, ^156^, ^157^] (*Tunable Aryl Alkyl Ionic Liquids*) such as [R(*n*Bu)Im]Br (R=2‐MePh, 4‐MePh, 2,4,6‐MePh=Mes, 2‐MeOPh, 2,4‐FPh, 4‐BrPh)^[145]^ to novel imidazolium hexacyanidosilicates (Scheme 13, middle). But only the combination with [BMIm]^+^ led to formation of a new IL, [BMIm]_2_[Si(CN)_6_] with *T*_m.p._=72 °C, which contains a double negatively charge anion.

**Figure 8:**
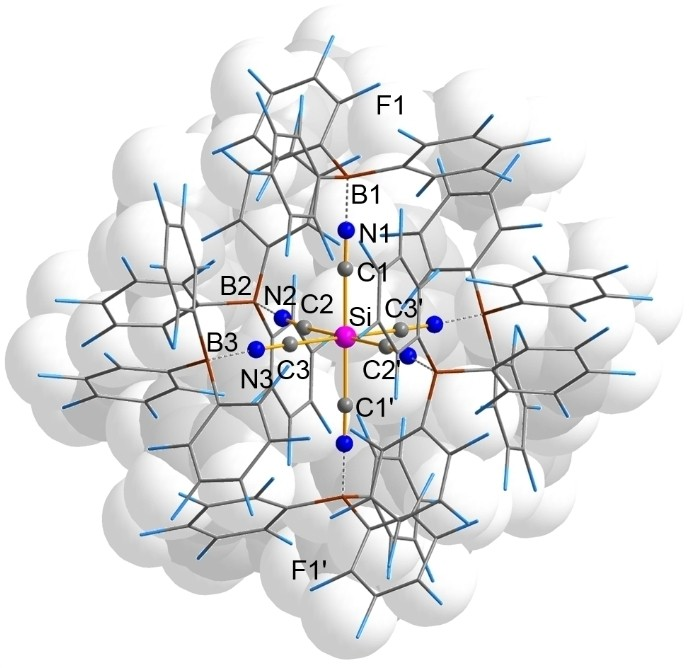
Representation of the molecular structure of the anion in the crystal of [Mes(*n*Bu)Im]_2_{Si[(CN)B(C_6_F_5_)_3_]_6_}. The [Si(CN)_6_] core is shown as ball‐and‐stick representation, while the B(C_6_F_5_)_3_ moieties are shown as wires‐and‐stick model. Cations and disorder are not shown for clarity. Colure code: Si pink, C grey, N blue, B brown,

In addition, the reaction of the hexacyanidosilcate dianion towards the Lewis acid tris(pentafluorophenyl)borane B(C_6_F_5_)_3_ was investigated.^[145]^ But since all synthesized ammonium and alkali metal salts were only soluble in Lewis basic solvents such as MeCN, MeOH or H_2_O, which react themselves with the borane, a cation was needed, which allowed the salt to dissolve in non‐Lewis basic solvents such as CH_2_Cl_2_. [Mes(*n*Bu)Im]_2_[Si(CN)_6_] now enabled the reaction with B(C_6_F_5_)_3_. Initially, we aimed to abstract one cyanide moiety affording in formation of [Si(CN)_5_]^−^ and [B(C_6_F_5_)_3_(CN)]^−^ when one equivalent of the borane is used. Unfortunately, only different hexacyanidosilicate‐borane substitution patterns could be observed by means of ^11^B{^1^H} NMR spectroscopy, but no compound could be isolated from the reaction mixture. But when the borane is used in excess (*n*>6), a complete functionalization of all cyanide ligands with the borane was found and the voluminous {Si[CN⋅B(C_6_F_5_)_3_]_6_}^2−^ adduct anion (*V*_anion_∼2.77 nm^3^) is formed (Scheme 13, bottom; Figure 8).

## 8 Conclusions

In conclusion, we have shown that salts containing the methyl carbonate anion like [R_3_MeE][CO_3_Me] (E=N, R=Et, *n*Pr or *n*Bu; E=P, R=Ph) are suitable starting materials for the quantitative synthesis of the corresponding ammonium and phosphonium pseudohalides [R_3_MeE]X (X^−^=CN, N_3_, OCN, SCN) as well as for hexafluorosilicate salts such as [R_3_MeN]_2_[SiF_6_] (R=*n*Pr, *n*Bu). Reactions of pseudohalides with Lewis‐acidic OP(OMe)_3_ and B(OMe)_3_ were investigated, resulting in formation of the [O_2_P(OMe)_2_]^−^ anion under methylation of the pseudohalide or the synthesis of the RT‐IL [*n*Bu_3_MeN][B(OMe)_3_(CN)], respectively. Reaction of the ILs [*n*Bu_3_MeN]X (X=CN, N_3_) with white phosphorus led to decomposition of the cation, yielding a free amine and polymeric phosphorus species. No novel anionic species could be extracted from the reaction of P_4_S_10_ with [*n*Bu_3_MeN]CN. When [WCC]CN ([WCC]=unsymmetrical) is dissolved in a slight excess of HCN, forming an IL, only fast polymerization of HCN was observed and no crystalline material could be obtained. With symmetrical cations, *e. g*. [PPh_4_] and [PPN], it was possible to isolate the elusive [CN(HCN)_2_]^−^ and [CN(HCN)_3_]^−^ anions from the IL‐like mixtures. We were able to isolate molecular [H(NCS)_2_]^−^, when treating [PPN]SCN with *in situ* generated HNCS. From an unusual crystallization medium (IL‐mixture+perfluoropolyether), we were able to generate and isolate [PPN][N_3_(HCN)_3_], [PPN][OCN(HCN)_3_], [PPN][SCN(HCN)_2_] as well as [PPN][P(CN⋅HCN)_2_], when treating [PPN]X (X=N_3_, OCN, SCN, OCP) with liquid HCN after adding perfluoropolyether. These are the first examples of solvate anions of the type [X(HY)_*n*_]^−^, which contain two different pseudohalides. We also managed to synthesize novel (cyanido)halidosilicates of the type [SiCl_0.78_(CN)_5.22_]^2−^, [SiF(CN)_5_]^2−^ and [Si(CN)_6_]^2−^ from temperature controlled halide/cyanide exchange reactions. Utilizing neutralization and salt metathesis reactions, we were able to obtain metal salts, such as Li_2_[Si(CN)_6_] ⋅ 2 H_2_O or K_2_[Si(CN)_6_] and novel imidazolium salts, respectively. Further, derivatization of the hexacyanidosilicate dianion with the Lewis acid B(C_6_F_5_)_3_ led to formation of the bulky adduct anion {Si[CN⋅B(C_6_F_5_)_3_]_6_}^2−^, which can be regarded as a very bulky WCA (weakly coordinating anion).

## Experimental Section

Caution! HCN and Me_3_Si−CN are highly toxic! Appropriate safety precautions (HCN detector, gas mask, low temperature) should be taken. All experimental data can be found in the original papers.

## Conflict of interest

The authors declare no conflict of interest.
